# Innate immune recognition of *Debaryomyces hansenii* requires Dectin-1-Card9 signaling

**DOI:** 10.1128/iai.00157-26

**Published:** 2026-08-04

**Authors:** Kevin P. Newhall, Sarah K. McNeer, Scott T. Espenschied, Elora G. Dolan, Julie Y. Zhou, Deborah A. Hogan, Thaddeus S. Stappenbeck

**Affiliations:** 1 Department of Inflammation and Immunity, Cleveland Clinic Research 2569, Cleveland, Ohio, USA; 2 Department of Pathology, Case Western Reserve University School of Medicine 12304, Cleveland, Ohio, USA; 3 Department of Microbiology and Immunology, Geisel School of Medicine at Dartmouth 12285 , Hanover, New Hampshire, USA; 4 Cleveland Clinic Lerner College of Medicine, Case Western Reserve University, Cleveland, Ohio, USA; University of Illinois Chicago, Chicago, Illinois, USA

**Keywords:** fungi, Dectin-1, phagocytosis, *D. hansenii*, myeloid cells

## Abstract

Innate immune signaling plays a key role in host response to infection, yet the pattern recognition receptors that detect non-model gut-associated yeasts remain poorly defined. Here, we investigated macrophage sensing of *Debaryomyces hansenii*, a food-derived yeast that we found to be enriched within intestinal ulcers of Crohn disease (CD) patients. Using a cell surface receptor antibody screen of bone marrow-derived macrophages infected with a CD patient isolate of *D. hansenii*, we showed that *D. hansenii*-induced macrophage activation characterized by increased expression of co-stimulatory molecules, MHC-II, and pattern recognition receptors, including the C-type lectin receptor Dectin-1. Antibody blockade experiments showed both Dectin-1 and complement receptor 3 subunit CD11b were required for phagocytosis of *D. hansenii*, while Dectin-1 was uniquely required for production of the pro-inflammatory cytokine tumor necrosis factor (Tnf). CRISPR-Cas9-mediated deletion of Dectin-1 phenocopied antibody neutralization effects on phagocytosis. Furthermore, deletion of Dectin-1 or its downstream signaling adaptor molecule Card9 resulted in reduced Tnf secretion in response to *D. hansenii*. Dectin-1-mediated uptake of *D. hansenii* was observed in primary bone marrow-derived macrophage and dendritic cells, as well as across the spectrum of macrophage polarization states. Together, these findings define the role of Dectin-1-Card9 signaling axis in innate immune cell sensing of *D. hansenii*. These findings support the emerging relevance of innate immune recognition of a yeast in Crohn disease pathogenesis.

## INTRODUCTION

Innate immune recognition of microbes is critical for mucosal homeostasis. In the intestine, ulcerations in the barrier can result in translocation of components of the microbiota into the underlying tissue, where immune cells, such as macrophages, rapidly sense and coordinate an immune response to this infection. Pattern recognition receptors (PRRs) detect broadly conserved microbial or damage-related molecular patterns and are critical mediators of the rapid macrophage response to infection. Numerous anti-fungal PRRs, including C-type lectin receptors (CLRs), recognize cell wall glycans present in various fungi (1). Together, these PRRs can induce phagocytosis, macrophage activation, and proinflammatory cytokine secretion to initiate inflammation. Furthermore, altered functions of the CLRs or their downstream effectors through human genetic polymorphisms or pharmacologic treatment are associated with increased risk of fungal infection (2–4) and chronic inflammatory diseases of the intestine (5–8).

The C-type lectin receptor (CLR) class of PRRs are cell membrane proteins that bind fungal cell wall glycans. Examples of which are β-glucan sensing by Dectin-1 (9, 10) and mannan sensing by Dectin-2 and Mincle (1). CLR engagement with ligands activates a downstream adaptor molecule Card9 that is required for subsequent NF-κB activation, which leads to proinflammatory cytokine expression, including tumor necrosis factor (Tnf) (11). Additionally, complement receptor 3 (CR3), which is composed of CD11b/CD18 heterodimers, can also directly engage β-glucan, independently of complement opsonization, and works with Dectin-1 to elicit innate immune responses (12–15).

Fungal intrinsic factors, such as individual species and state-specific cell wall composition, dictate which PRRs will be engaged. For example, Dectin-1 senses germinating, but not resting, *Aspergillus fumigatus* conidia (16). The dimorphic fungus *Candida albicans* induces Dectin-1 sensing predominantly by yeast cells (17) and Dectin-2 sensing by hyphae (18). Overall, these studies highlight that PRR sensing of individual species of fungi is highly context-dependent, underscoring the need for defining mechanisms of PRR sensing for individual species.

There is increasing evidence for the role of fungi in chronic inflammatory diseases of the intestine, such as Crohn disease (CD) (7, 19–26). One such yeast, *Debaryomyces hansenii*, is predominantly a food microbe present in cheeses and processed meats and is generally recognized as safe for human consumption (27, 28). Recently, our group identified culturable *D. hansenii* within the ulcerated regions of the intestine in CD patients (19). Introduction of human CD *D. hansenii* isolates into mice with an intestinal injury leads to delayed wound repair through a macrophage Tlr3-Type I IFN-Ccl5-dependent mechanism. Furthermore, fluorescent-activated cell sorting analysis of intestinal wounds identified live *D. hansenii* within the CD45+ F4/80+ cell compartment, consistent with intestinal phagocytes, such as macrophages, in regulating the immune response to fungal infection (19). While the role of the intracellular PRR Tlr3 in mediating host response to *D. hansenii* is known, the repertoire of PRRs, which mediate phagocytosis and delivery of the yeast to the phagosome to permit Tlr3-ligand interactions, remains unknown. Our current opportunity is to define mechanisms of macrophage recognition and uptake of patient strains of *D. hansenii* using *in vitro* systems.

In the present study, we aimed to determine macrophage phagocytosis of and activation in response to a CD-patient strain of *D. hansenii*. To do this, we utilized a candidate antibody-based flow cytometry screen to identify differentially abundant cell surface immune molecules following *D. hansenii* infection of bone marrow-derived macrophages (BMDMs). Alongside other classical macrophage activation markers, we identified Dectin-1 as being significantly upregulated following *D. hansenii* infection. Using fluorescently labeled strains of *D. hansenii* in combination with reverse genetic and antibody blockade approaches, we determined that Dectin-1- and CD11b-mediated phagocytosis of *D. hansenii*. Signaling through Dectin-1 and Card9 results in Tnf induction following *D. hansenii* engagement. Taken together, these data elucidate the mechanisms by which macrophages sense and respond to an emerging fungus of biomedical interest for patients with chronic inflammatory diseases of the intestine such as CD.

## RESULTS

### BMDM activation in response to *D. hansenii* infection

**Fig 1:**
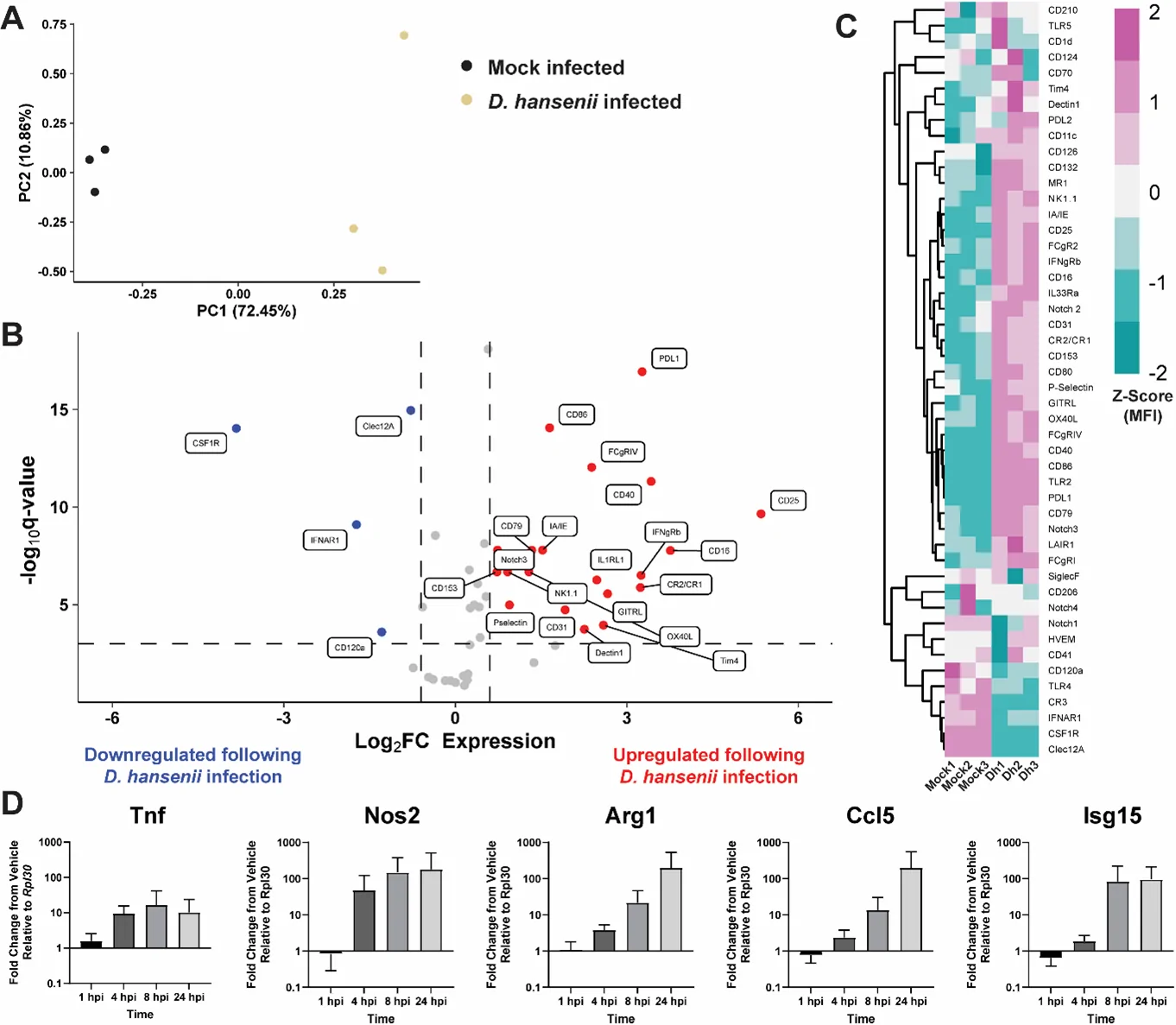
*D. hansenii* induces bone marrow-derived macrophage (BMDM) activation. (**A**) Principal component analysis of MFI. (**B**) Volcano plot of differentially expressed cell surface proteins 16 hpi with *D. hansenii* CDA1. *X*-axis is log_2_(fold change) in MFI relative to mock-infected, *Y*-axis is −log(*q*-value). (**C**) Heatmap of median fluorescence intensity (MFI) *Z*-score of indicated cell surface receptor marker on BMDM detected through flow cytometry. “Mock” denotes vehicle-treated mock infection; “dh” denotes *D. hansenii* infection. *N* = 3 replicates from one experiment. (**D**) RT-qPCR at indicated timepoints of BMDMs infected with *D. hansenii* strain CDA1. *N* = 6 replicates from three independent experiments. Error bars denote standard deviation. Statistical analysis: One-way analysis of variance. Tnf: *F*(3, 20) = 1.089, *P* = 0.3765; Nos2: *F*(3, 20) = 1.022, *P* = 0.4038; Arg1: *F*(3, 20) = 1.976, *P* = 0.1500; Ccl5: *F*(3, 20) = 1.852, *P* = 0.1703; Isg15: *F*(3, 20) = 1.917, *P* = 0.1594.

Given the prior observations that (i) F4/80+ cells in the intestine co-localized with *D. hansenii* and (ii) live *D. hansenii* could be recovered from purified intestinal CD45+/F4/80+ phagocytes, we initially modeled macrophage interactions with *D. hansenii in vitro* using bone marrow-derived macrophages (BMDMs). Following 16-h infection with *D. hansenii*, we performed a targeted flow cytometry-based screen of cell surface receptors using a fluorescently conjugated antibody panel to characterize macrophage responses to *D. hansenii*. We quantified expression (median fluorescence intensity, MFI) of 48 cell surface immune-related molecules (Table S1). Principal component analysis showed marked changes in BMDM cell surface receptors following *D. hansenii* infection relative to mock-infected (vehicle-treated) cells, with PC1 separating samples by treatment and accounting for 72% of the variance (Fig. 1A). Relative to mock-infected cells, *D. hansenii* infection resulted in 20 significantly upregulated and 4 significantly downregulated proteins, determined via MFI, which passed cutoff for significance (*q* < 0.05) and fold change (1.5) from uninfected cells (Fig. 1B and C). We also noted the co-stimulatory molecules CD40, CD86, and OX40L, and the antigen presentation molecule MHC-II (IA/IE) were upregulated at 16 h post*-D. hansenii* infection, consistent with macrophage activation following microbial infection (29).

Tumor necrosis factor (Tnf) is a proinflammatory cytokine secreted by activated macrophages that supports clearance of microbes by these cells (30, 31). Using time course gene expression analysis by reverse transcription quantitative PCR (RT-qPCR), we observed induction of Tnf relative to mock-infected BMDMs as early as 1 and 4 h post infection (hpi) (Fig. 1D). We also observed Nos2 induction at 4 hpi that was maintained over 24 h, while Arg1 showed slower induction kinetics that trailed behind Nos2. These data are consistent with early proinflammatory activation of BMDMs.

*D. hansenii* is known to induce a Type I IFN-Ccl5 response through Tlr3 in BMDMs, and this signaling axis is required for impaired regeneration following intestinal injury *in vivo* (19). We validated this in an *in vitro* model, noting marked upregulation of interferon stimulated gene 15 (*Isg15*) and *Ccl5* following infection (Fig. 1D). Interestingly, we also noted reduced MFI for Ifnar1 in *D. hansenii*-infected BMDMs, consistent with ligand-induced downregulation following activation (32). These data indicate that modeling macrophage interactions with *D. hansenii* induces BMDM activation toward an antimicrobial phenotype, with conservation of key pathways known to block intestinal injury repair *in vivo* (viz., Type I IFN signaling).

We sought to determine the dynamics of non-Tlr3 PRRs in macrophages following *D. hansenii* infection. We observed increased expression of the PRRs Dectin-1, CR2/CR1, and FcgRIV at 16 hpi (Fig. 1B and C). We also observed CD11b (CR3) trending toward downregulation but not reaching significance. Notably, CD11b is constitutively highly expressed by macrophages, while Dectin-1 is generally lowly expressed at baseline. The observed changes are likely related to post-activation transcriptional responses manifesting at the protein level.

### Generation of fluorescent reporter strains of *D. hansenii*

**Fig 2:**
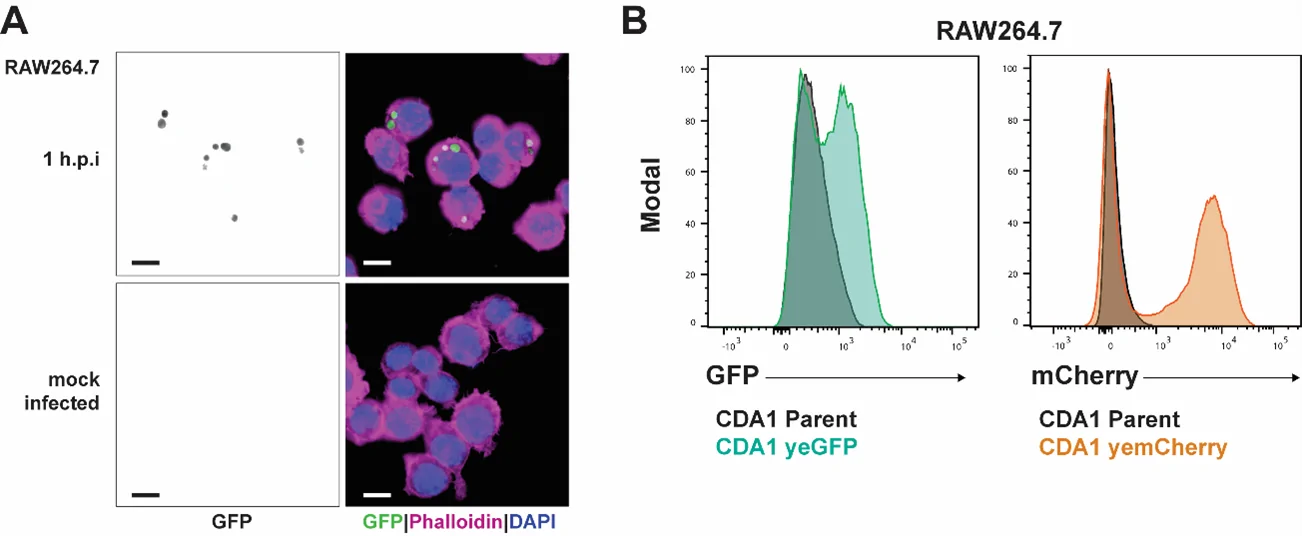
Fluorescent reporter strains of *D. hansenii* can be detected within macrophages. (**A**) Fluorescent micrograph of RAW 264.7 macrophage-like cell line infected with *D. hansenii* CDC1-yeGFP for 1 h. Left, isolated GFP channel inverted to highlight individual yeast cells. Right, composite image of DAPI (blue), phalloidin (magenta), and GFP (green) channels. Bars = 10 µm. (**B**) Flow cytometry histogram of RAW 264.7 macrophages infected with *D. hansenii* untransformed CDA1 parental strain (black), CDA1-yeGFP (teal), or CDA1-yemCherry (orange) strains.

Following engagement of pattern recognition receptors, macrophages are capable of phagocytosis to uptake the foreign material. The utilization of fluorescent reporter strains of yeast facilitates studies characterizing phagocytosis of these fungi (33). We generated fluorescent strains of *D. hansenii* to monitor phagocytosis and characterize the PRRs that mediate uptake. We stably transformed *D. hansenii* and selected for lines that expressed yeast-enhanced mCherry (yemCherry) or yeast-enhanced green fluorescent protein (yeGFP) (34). Using the model macrophage-like cell line RAW 264.7, we observed transformed *D. hansenii* strains within macrophages using fluorescent microscopy and flow cytometry (Fig. 2A and B). We observed increased signal-to-noise sensitivity using yemCherry-labeled *D. hansenii* strains as opposed to yeGFP. These engineered yeast strains could then be used for experiments to identify factors that mediate *D. hansenii* uptake by phagocytes.

### Dectin-1 and CD11b mediate *D. hansenii* phagocytosis

We next determined the cell surface receptors that functionally mediate uptake of *D. hansenii*. We hypothesized that pre-treatment of RAW 264.7 cells with candidate PRR blocking antibodies would reduce macrophage uptake of *D. hansenii*. Following infection (multiplicity of infection of 1 yeast per macrophage) for 1 h, we stained cells with an anti-CD45 antibody then performed analytical flow cytometry. This permitted quantification of the proportion of CD45+ yemCherry+ population (macrophage with yeast) relative to the total CD45+ population (macrophage alone).

**Fig 3:**
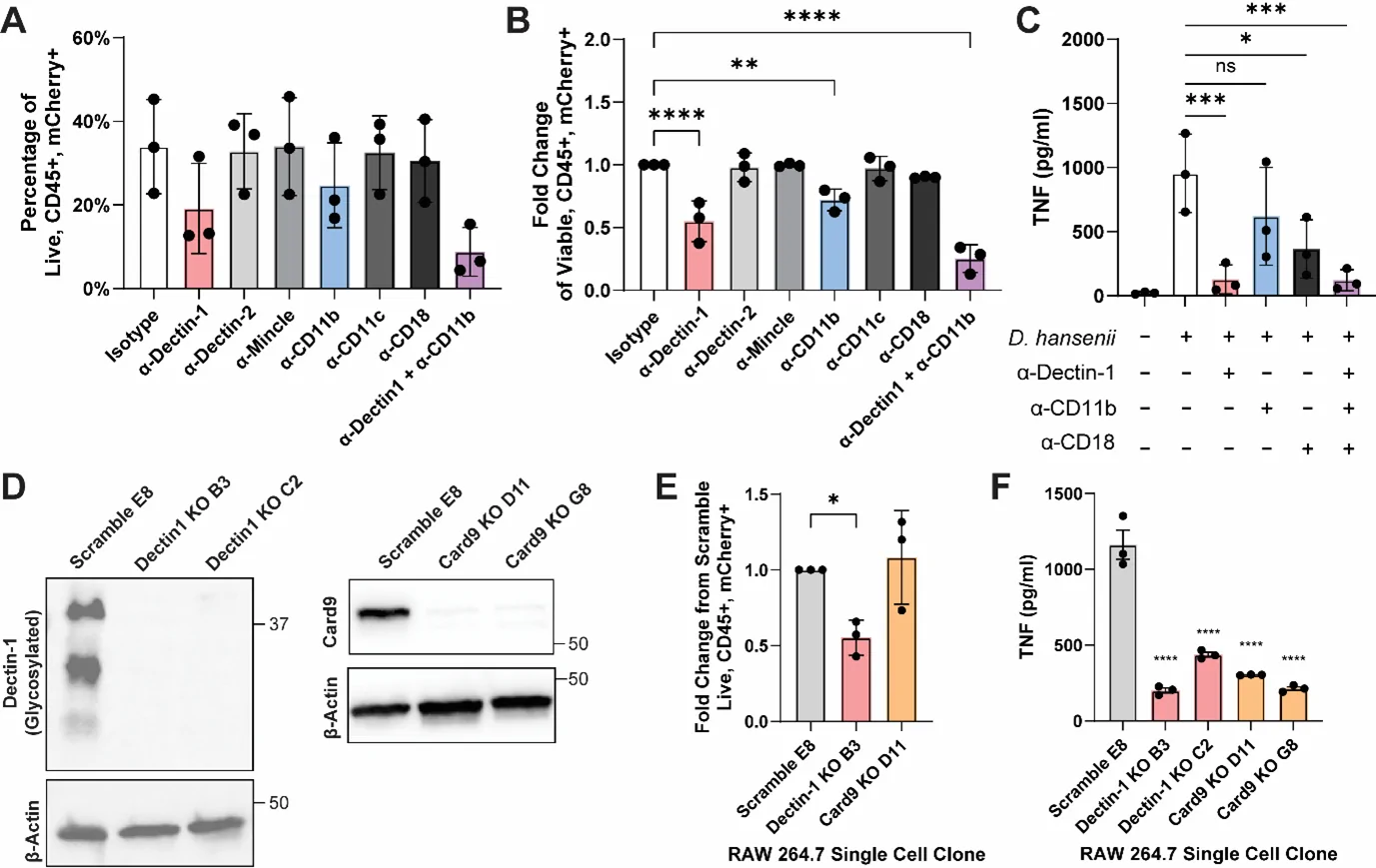
Dectin-1 is a major pattern recognition receptor of *D. hansenii* in RAW 264.7 cells. (**A**) Percentage of live, CD45+, mCherry+ RAW 264.7 cells following preincubation with indicated cell surface molecule blocking antibody and infection for 1 h with *D. hansenii* CDA1-yemCherry. *N* = 3 independent experiments. (**B**) Fold change from isotype control-treated cells as shown in panel A. (**C**) Supernatant Tnf concentrations were detected via enzyme-linked immunosorbent assay from RAW 264.7 cells after 4 h infection with *D. hansenii* CDA1. *N* = 3 independent experiments. (**D**) Immunoblot validation of CRISPR-Cas9-generated knockout single-cell clone lines. Dectin-1 exhibits bands of multiple sizes due to glycosylation. (**E**) Fold change in live, CD45+ mCherry+ RAW 264.7 cells relative to negative control scramble clone E8. *N* = 3 independent experiments. (**F**) Supernatant Tnf concentrations from indicated clones infected with CDA1 for 4 h. Representative experiment of three independent experiments with similar results. Statistical analysis: * *P* < 0.05, **P < 0.01, ***P < 0.001, ****P < 0.0001. (**A**) One-way analysis of variance *F*(7, 16) = 2.540, *P* = 0.0581. (**B**) One-way analysis of variance, *F*(8, 18) = 53.43, *P* < 0.0001. (**C**) One-way analysis of variance, *F*(7, 16) = 7.557, *P* = 0.0004, Dunnett’s multiple comparisons test. (**E**) One-way analysis of variance, *F*(2, 6) = 6.705, *P* = 0.0295, Dunnett’s multiple comparisons test. (**F**) One-way analysis of variance: *F*(4, 10) = 80.55 *P* < 0.0001, Dunnett’s multiple comparisons test.

Using these conditions, ~35% of isotype antibody-treated cells contained *D. hansenii*-mCherry signal (Fig. 3A). However, following blockade of candidate CLRs and components of the complement receptor, we detected reductions in the proportion of CD45+ yemCherry+ cells (Fig. 3B). Notably, Dectin-1 blockade resulted in a ~50% reduction in the proportion of phagocytosis-positive macrophages, while blockade of other fungal sensing PRRs Dectin2 and Mincle was not associated with a reduction in the percentage of phagocytosis-positive macrophages. Furthermore, we observed a ~25% reduction following blockade of CD11b, a component of complement receptor 3, but did not observe a significant change following blockade with CD11c or CD18. Blockade of both Dectin-1 and CD11b had an additive effect, resulting in a ~75% reduction in phagocytosis.

### Dectin-1 and Card9 mediate Tnf secretion in response to *D. hansenii*

In addition to mediating uptake, Dectin-1 can potentiate proinflammatory cytokine secretion (35). Blockade of Dectin-1 markedly reduced the concentration of Tnf in the media at four hpi (Fig. 3C). Blockade of the components of CR3 alongside Dectin-1 did not further reduce TNF secretion. These data indicate that Dectin-1 is the major PRR that mediates Tnf secretion in RAW 264.7 cells following *D. hansenii* infection.

To orthogonally validate the role of Dectin-1 in phagocytosis and Tnf induction in response to *D. hansenii*, we generated Dectin-1 and Card9 KO RAW 264.7 cells using CRISPR-Cas9 mutagenesis and single-cell cloning (Fig. 3D). Scramble gRNA transduced cells served as a control. We observed Dectin-1 KO cells exhibited a 50% reduction in phagocytosis of *D. hansenii* relative to the scramble control line (Fig. 3E). Cells deficient in the downstream adaptor for Dectin-1 signaling, Card9, did not display any alterations to phagocytosis relative to the control. These data further strengthen that Dectin-1 is a major phagocytosis receptor of *D. hansenii*.

The PRR Dectin-1 signals through the adaptor protein Card9 to induce proinflammatory cytokine secretion such as Tnf (35). As we had previously observed, an increase in Tnf transcript began at 1 and 4 hpi (Fig. 1B); we measured secreted Tnf at 4 hpi to study the response downstream of PRR sensing via Dectin-1. Using multiple single-cell clones, we showed that Dectin-1 KO and Card9 KO RAW 264.7 cells produced significantly less Tnf at 4 hpi with *D. hansenii* as compared to the non-targeting scramble gRNA controls (Fig. 3F). These data indicate that the Dectin-1-Card9 signaling axis supports Tnf secretion by macrophages following *D. hansenii* engagement.

### Primary macrophages and dendritic cells utilize Dectin-1 to mediate *D. hansenii* uptake

**Fig 4:**
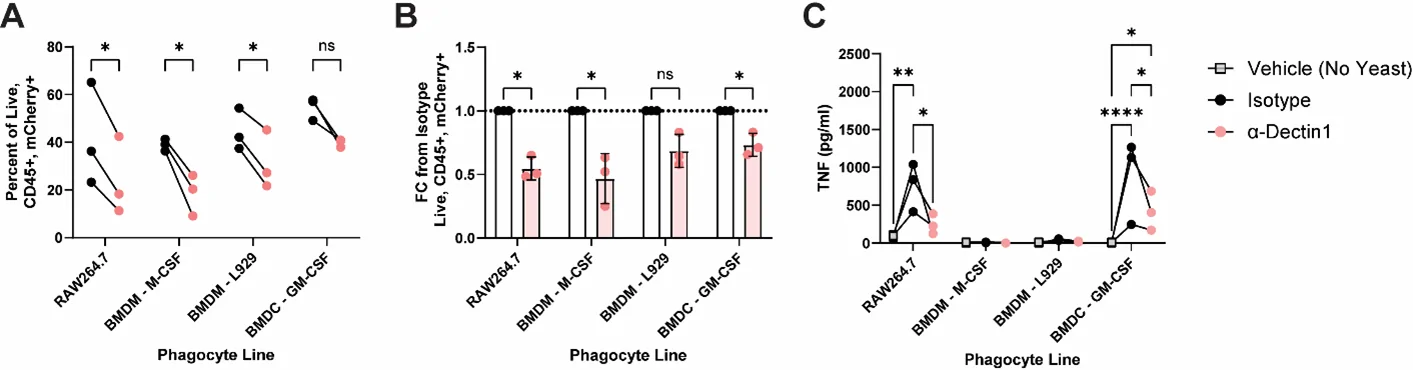
Bone marrow-derived macrophages and dendritic cells utilize Dectin-1 to uptake CDA1. (**A**) Flow cytometric quantification of *D. hansenii* CDA1-yemCherry phagocytosis by indicated phagocytes. *N* = 3 independent experiments. (**B**) Fold change in mCherry+ cells relative to isotype control for indicated phagocytes as shown in A. (**C**) Enzyme-linked immunosorbent assay quantification of supernatant TNF at 4 h post-infection. *N* = 3 independent experiments. Statistical analysis: * denotes *P* < 0.05, ** denotes *P* < 0.01, **** denotes *P* < 0.0001. (**A**) Paired *t*-test. RAW 264.7, *T*(2) = 5.629, *P* = 0.030; BMDM-M-CSF, *T*(2) = 5.666, *P* = 0.029; BMDM-L929, *T*(2) = 6.443, *P* = 0.023; BMDC-GM-CSF, *T*(2) = 4.298, *P* = 0.050. (**B**) Paired *t*-test. RAW 264.7, *T*(2) = 8.681, *P* = 0.013; BMDM-M-CSF, *T*(2) = 4.690, *P* = 0.042; BMDM-L929, *T*(2) = 4.180, *P* = 0.052; BMDC-GM-CSF, *T*(2) = 5.112, *P* = 0.036. (**C**) Two-way analysis of variance by treatment *F*(2, 24) = 11.71, *P* = 0.0003. Tukey’s multiple comparisons *post hoc* test.

Dectin-1 participates in uptake in diverse phagocyte lineages, though its expression varies across cell types (35, 36). To further assess the contribution of Dectin-1 in *D. hansenii* uptake, we performed phagocytosis assays without and with Dectin-1-blocking antibody in primary BMDMs differentiated via either recombinant macrophage colony-stimulating factor (M-CSF)- or L929-conditioned media (CM), as well as primary bone marrow-derived dendritic cells (BMDCs) differentiated with recombinant granulocyte-macrophage colony-stimulating factor (GM-CSF). Dectin-1 blockade led to a significant reduction in fungal uptake by ~50% in M-CSF-differentiated BMDMs, similar to levels of phagocytosis in RAW 264.7 cells. Uptake of *D. hansenii* was reduced by Dectin-1 blockade by ~25% in L929-CM-treated BMDMs (*P* < 0.05) with a similar trend in GM-CSF-treated BMDCs, though the latter difference was not significant (Fig. 4A and B).

Dectin-1 agonism in BMDCs but not BMDMs induces Tnf secretion (35). While both BMDCs and BMDMs phagocytosed *D. hansenii* in part through Dectin-1 (Fig. 4A and B), only BMDCs secreted Tnf upon exposure to fungal cells to levels similar to what was observed for RAW 264.7 cells, and Dectin-1 blockade via neutralizing antibody reduced Tnf release into the media. M-CSF- and L929-CM-differentiated BMDMs did not release Tnf in response to *D. hansenii* (Fig. 4C). These data indicate that, like other fungi and pure Dectin-1 agonism, BMDCs elicit a robust Tnf response in response to *D. hansenii*, but BMDMs fail to do so.

**Fig 5:**
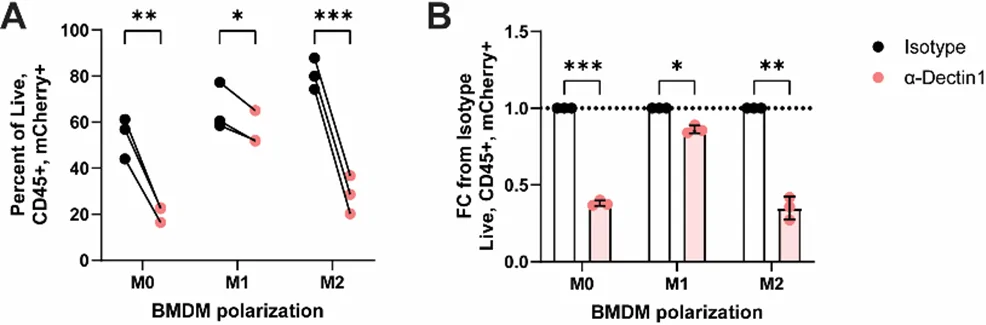
Dectin-1 mediated uptake of *D. hansenii* varies between macrophage polarization states. (**A**) Flow cytometric quantification of *D. hansenii* CDA1-yemCherry phagocytosis by M-CSF-differentiated BMDMs unpolarized (M0) or polarized to proinflammatory (M1) or anti-inflammatory (M2) macrophage. (**B**) Fold change in mCherry+ cells relative to isotype control. Statistical analysis: * denotes *P* < 0.05, ** denotes *P* < 0.01, *** denotes *P* < 0.001. (**A**) Paired *t*-test. M0, *T*(2) = 10.34, *P* = 0.009; M1, *T*(2) = 5.339, *P* = 0.033; M2, *T*(2) = 53.97, *P* = 0.0003. (**B**) Paired *t*-test. M0, *T*(2) = 59.17, *P* = 0.0002; M1, T(2) = 9.365, *P* = 0.011; M2, *T*(2) = 15.22, *P* = 0.004.

As M-CSF-differentiated BMDMs typically exist in an unpolarized state (M0), we next polarized BMDMs toward a proinflammatory state with IFN-γ and LPS (M1), or an anti-inflammatory state with IL-4 (M2) (37). We observed Dectin-1 neutralization kinetics varied between macrophage polarization states (Fig. 5A and B). When treated with Dectin-1 neutralizing antibody, we observed M1s were less dependent on Dectin-1 to mediate phagocytosis. Dectin-1 blockade reduced uptake in M0 and M2 macrophages, however. These data indicate that Dectin-1 utilization varies between macrophage polarization states and thus modulates *D. hansenii* uptake.

## DISCUSSION

Numerous PRRs respond to non-self-microbial products, but only a subset is relevant in the immune response to each microbial species (1). Our goal was to model macrophage-*D. hansenii* interactions *in vitro* to define the repertoire of PRRs that mediate macrophage activation and phagocytosis. We showed that BMDMs infected with *D. hansenii* undergo proinflammatory activation, including upregulation of co-stimulation markers, MHC class II, and select PRRs such as Dectin-1. Using genetically modified fluorescent reporter strains of *D. hansenii* generated herein, we identified that Dectin-1 and CD11b are critical PRRs that mediate phagocytosis of *D. hansenii*. Other candidate PRRs, including Dectin-2, Mincle, and CD11c, are not required for phagocytosis of *D. hansenii*. Mechanistically, we showed that a Dectin-1-Card9 signaling axis is required to induce Tnf in response to *D. hansenii*. We validate the role of Dectin-1 in phagocytosis of *D. hansenii* in primary BMDM and BMDCs, as well as polarized BMDMs. Altogether, these data contribute increased understanding as to the identities of candidate PRRs that mediate sensing of *D. hansenii*.

*In vivo*, *D. hansenii* co-localizes with F4/80+ cells and blocks epithelial injury repair in a Tlr3-Type I IFN-Ccl5-dependent manner. Notably, we previously showed that Dectin-1^−/−^ BMDMs do not exhibit a defect in Ccl5 production (19). Here, we demonstrate that multiple PRRs, including Dectin-1 and CD11b, mediate phagocytosis of *D. hansenii*. Based on these results, one interpretation is that redundancy through CD11b could provide a secondary mechanism for uptake and delivery of yeast to Tlr3 containing phagosomes. Indeed, others have observed Dectin-1 and CR3 redundancy in mediating fungal uptake (15). Furthermore, *Dectin-1*^−/−^ mice exhibit significant facility- and microbiota-dependent effects on colitis, with some studies identifying protective effects and others exhibiting exacerbation due to Dectin-1 deficiency (25, 38, 39).

The Dectin-1-Card9-Tnf signaling axis is variable between primary mouse myeloid cell types *in vitro* (35, 40). We observed a lack of secreted Tnf protein in M-CSF-differentiated BMDMs stimulated with *D. hansenii* at 4 h post infection. Other fungal pathogens, including *Candida albicans*, *Malassezia restricta*, and *Malassezia globosa*, similarly failed to induce Tnf secretion from M-CSF-differentiated BMDMs, while GM-CSF-differentiated BMDCs were capable of inducing secreted Tnf (40). Stimulating BMDMs with additional signals including IL-3, GM-CSF, or IFN-γ supports Tnf secretion through Dectin-1-Card9 signaling (35, 41).

Although Dectin-1-Card9 blockade significantly reduced RAW 264.7 and BMDC Tnf secretion in response to *D. hansenii*, there was still residual Tnf induction; this indicates that additional cell surface receptors may contribute to *D. hansenii* sensing. Numerous PRRs may be responsible and likely in a cell type-specific manner. For instance, Tlr2 is known to synergize with Dectin-1 to induce TNF in response to *C. albicans* or zymosan (42, 43). Future studies will be required to determine the extent to which Tlr2, or other PRRs upstream of Myd88, contribute to the sensing of *D. hansenii*, and if Dectin-1-Tlr2 coactivation affects downstream cytokine responses.

Our cell surface receptor screen identified additional proteins with altered expression in *D. hansenii*-infected BMDMs that warrant further study. Notably, PD-L1 showed increased expression following 16 h of infection with *D. hansenii*. PD-L1 is most classically known to be an immune checkpoint molecule but more recently shown to directly recognize numerous fungi including *Saccharomyces cerevisiae*, *C. albicans*, and more (44). In neutrophils, a Dectin-1-Card9 axis supports upregulation of PD-L1 after 8 h of infection (45). Whether PD-L1 upregulation in *D. hansenii*-infected macrophages serves as a PRR-mediating fungal sensing, or an immune checkpoint molecule tuning immunity, is an interesting avenue of further investigation that may better help us understand *in vivo* infection dynamics to this microbe.

Human CD patients show *D. hansenii*-reactive adaptive immune responses including T-cell responses and serum immunoglobulin targeting *D. hansenii* (19, 20). In mouse models, a specific population of Card9+ CX3CR1+ antigen presenting cells are required for generating adaptive immune responses including serum antibodies that target gut fungi independent of colitis (46). Additional work profiling the role of a Dectin-1-Card9 axis in inducing adaptive immune activation, including T-cell polarization and antibody class switching, would be of benefit to determine how Dectin-1 sensing by antigen presenting cells skews adaptive immunity to *D. hansenii*. The ability to modulate phagocytosis of *D. hansenii* in these experimental systems will be a valuable tool to better understand the bridge from innate to adaptive immunity to *D. hansenii* in the context of intestinal inflammation.

## MATERIALS AND METHODS

### Yeast culture

*D. hansenii* strains CDA1 and CDC1 were previously obtained from Crohn disease patients (19). Strains were routinely cultured on Sabouraud’s Dextrose Agar (Sigma 84088) or Yeast Peptone Dextrose (YPD) agar at 30°C. For infection assays, strains were cultured overnight at 30°C in Yeast Peptone Dextrose (YPD) medium. Cells were washed and resuspended in sterile phosphate-buffered saline (PBS) before infection.

### Cell line culture

RAW 264.7 (ATCC TIB-71) cells were obtained from the Cleveland Clinic Cell Services Core. L929 cells were obtained from ATCC (#CCL-1). Cells were maintained in DMEM supplemented with 10% Fetal Bovine Serum (Sigma F4135) and 1% penicillin/streptomycin (complete DMEM).

L929-conditioned medium was generated by allowing six 150 cm flasks to grow to confluence in complete DMEM. Cells were rinsed with PBS, then supplemented with 25 mL of fresh complete DMEM. After 24 h, conditioned medium was collected, centrifuged at 3,000 rpm for 5 min, pooled, and stored at 4°C. This was repeated for four total collections, after which pooled media was aliquoted and stored at −20°C until use for bone marrow differentiation.

### Bone marrow-derived macrophages and dendritic cells

C57BL/6J mice were obtained from the Jackson Laboratory. Bone marrow was harvested from mice at greater than 8 weeks of age. Mice were housed under specific-pathogen-free conditions. All animal studies were conducted in compliance with protocols approved by the Cleveland Clinic Institutional Animal Care and Use Committee.

Mice were euthanized via CO_2_ inhalation and secondary cervical dislocation. Long bones were harvested from the hind legs, and bone marrow was isolated. Cells were strained with a 70 μm filter. For cytokine differentiated BMDMs and BMDCs, red blood cells were lysed in ACK lysing buffer (ThermoFisher), and remaining bone marrow cells were counted via hemacytometer. Cells were plated at 2 × 10^6^ cells in a 10 cm tissue cultured treated dish in 10 mL of differentiation medium (10% fetal bovine serum (FBS), 1% penicillin/streptomycin, and recombinant cytokines in RPMI). For bone marrow-derived macrophages, 25 ng/mL of recombinant M-CSF (Peprotech 315-02) was used. For bone marrow dendritic cells, 25 ng/mL of recombinant GM-CSF (Peprotech 315-03) was used. Every 2 days, medium was siphoned off of plates, centrifuged to pellet non-adherent cells, and replated in 10 mL of fresh medium. On day 7 after differentiation, cells were plated in tissue culture-treated 24-well plates at 2 × 10^5^ cells per well in complete RPMI without differentiation cytokines. Where indicated, M-CSF-differentiated BMDMs were polarized for 20 h with 20 ng/mL of LPS (Invivogen, tlrl-eblps) and 50 ng/mL of IFNy (BioLegend 575304) (M1) or 20 ng/mL IL-4 (BioLegend 574304) (M2) prior to infection. Polarization was validated using Nos2 and Arg1 RT-qPCR. For L929-differentiated bone marrow macrophages, marrow was harvested from long bones, counted, and 5 × 10^6^ total cells without RBC lysis were differentiated in 10 mL of complete RPMI supplemented with 25% L929 CM. After 3 days, 3 mL of fresh media were added. On day 6, cells received a full 10 mL media change before plating on day 7. Validation of differentiation with 25% L929-conditioned medium resulted in 96% of live, single cells being F4/80+ CD11b+.

### Cell surface antibody screen

Antibodies utilized in the screen are listed in Table S1. One microliter of indicated PE-conjugated antibody was added to each well in technical triplicate along with 19 μL of MACS buffer (PBS, 2mM EDTA, 0.5% FBS). L929 BMDMs were generated as described above in 10 cm dishes. Overnight culture of *D. hansenii* CDA1 was grown in liquid YPD. Yeast cells were washed, counted, and resuspended in sterile water. Four dishes per condition were infected with 10 yeast cells per macrophage in sterile water or vehicle sterile water alone (mock-infected) for 16 h. BMDMs were washed using PBS and scraped in MACS buffer (PBS, 2 mM EDTA, 0.5% FBS) to harvest. Cells were pooled, counted, and treated with TruStain FcX (BioLegend 101320) for 5 min on ice. Cells were stained with FITC-conjugated anti-F4/80 (BioLegend 123108) for 5 min on ice. Prepared BMDMs (1 × 10^5^) were aliquoted into each well of a 96-well plate and triturated. Cells were stained for 15 min on ice before washing and resuspending in MACS buffer with Sytox Blue viability dye (Thermo Fisher). Flow cytometry was performed using Thermo Fisher Attune NxT with autosampler. Single-color F4/80-FITC, PE-isotype, and Sytox Blue-stained cells were used for compensation.

Data were analyzed using FlowJo v10.8. PE MFI was determined by gating for single, Sytox−, F4/80+ cells. In the case of a negative value for the MFI due to compensation, the most negative value for that antibody was added to each technical replicate for that antibody across conditions to generate a non-negative value. Multiple unpaired *t*-tests were corrected for multiple comparisons using false discovery rate correction (*Q* = 1.00%) using the Benjamini, Krieger, and Yekutieli method to determine a *q*-value for significance. Statistical analysis was performed using GraphPad Prism 10.6.1 and R (version 4.4.0 [2024-04-24]) and the following packages: pheatmap, ggplot2, ggrepel, and NatParksPalettes.

### Reverse transcription quantitative PCR

RNA was isolated from cells using the Qiagen RNeasy Plus Mini Kit (74134) following manufacturer’s instructions. In brief, following treatment with *D. hansenii* at a multiplicity of infection of 10 yeast per macrophage, macrophages were washed with PBS and lysed using Qiagen RLT buffer supplemented with 2-mercaptoethanol per manufacturer’s instructions. Cells were scraped off the well bottom with a pipet tip and frozen at −80°C until RNA extraction. RNA was eluted in molecular biology-grade water and concentration was quantified using Cytation 5 Nanoquant plate. cDNA was synthesized using iScript cDNA synthesis kit (Bio-Rad, #1708890) and qPCR was performed using 10 ng of cDNA in a 20 μL reaction with TB-Green Advantage qPCR master mix (Takara #639676) using a Bio-Rad CFX Connect Real-Time PCR detection system. Fold change was calculated using 2^−ΔΔCt^ relative to mock-infected (vehicle-treated) cells at each timepoint using the reference gene Rpl30 (47). The following primers were used: Ccl5 (F: TGCCCACGTCAAGGAGTATTTC; R: AACCCACTTCTTCTCTGGGTTG), Isg15 (F: AAGCTCAGCCAGAACTGGTCT; R: ATGGCCTGGGACCTAAAGGTGAA), Tnf (F: CAAAGGGATGAGAAGTTCCC; R: CTTGGTGGTTTGCTACGACG), Nos2 (F: GCTCCTTTAAAGAGGCAA; R: TCAAAGACCTGCAGGTTG), Arg1 (F: CAAAAGGACAGCCTCGA; R: CGTGGGTTCTTCACAATTTG), Rpl30 (F: CCAGCTTTGAGGAAATCTG; R: GATAGCCAGTGTGCATAC).

### Generation of fluorescently labeled *D. hansenii* strains

Plasmids pAYCU268 and pAYCU257 were a kind gift of Nicholas Papon (34). pAYCU268 contains yeast-enhanced GFP and nourseothricin resistance. pAYCU257 encodes yeast-enhanced mCherry and mycophenolic acid resistance cassette. mCherry was cloned from pAYCU257 into pAYCU268 using restriction enzyme digest with BamHI-HF (NEB #R3136S) and AvrII (NEB #R0174S) and subsequent Quick Ligation (NEB #M2200) to swap fluorescent reporter genes. Plasmid sequences were confirmed with whole plasmid sequencing from Eurofins Genomics. Insertion cassettes were generated by digesting plasmids with ApaI (NEB #R0114S) and NsiI-HF (NEB #R3127L) and purified using the Monarch Spin DNA Gel Extraction kit and eluted in water (NEB #T1120S).

Electrocompetent *D. hansenii* cells for transformation were generated as described elsewhere (48). In brief, 50 mL cultures were grown overnight and harvested at an OD_600_ of 1–2. Cells were washed with distilled water and resuspended in 10 mL of transformation buffer (10 mM Tris, 1 mM EDTA pH 8.0, and 0.1 M lithium acetate) and shaken at 150 rpm at 30°C for 1 h. Dithiothreitol was added to a final concentration of 25 mM and shaken for an additional 30 min. Cells were washed with ice-cold distilled water twice and once with ice-cold 1 M sorbitol before resuspending the pellet in 200 μL of 1 M ice-cold sorbitol. Forty microliters of cell suspension were incubated with 1.5–2 μg of DNA for 5 min on ice before electroporating in a 0.2 cm cuvette with a Bio-Rad GenePulser X-cell at 2,300 volts, 25 μF, and 100 ohms. Following shock delivery, cuvettes were flooded with 1 mL of YPD medium and recovered by shaking at 30°C at 150 rpm for 4 h. Cells were spread on Sabouraud’s Dextrose Agar plates supplemented with 200 μg/mL of Nourseothricin (GoldBio #N-500-2) at 30°C until colonies formed. Cells were validated for fluorescent protein expression using Bio-Rad Chemidoc with fluorescence imaging and flow cytometry.

### Fluorescent microscopy

RAW 264.7 cells were seeded onto uncoated cover glass and allowed to adhere overnight before infection with *D. hansenii* for 1 h with an MOI of 10 yeast per macrophage or mock-infected. Cells were washed with PBS then fixed for 15 min in 4% PFA. Cells were washed with PBS and permeabilized with 0.1% Triton-X in PBS for 15 min. Cells were stained for 90 min with phalloidin Alexa Fluor 594 (Thermo Fisher A12381) diluted 1:400. Cells were washed 3 × 5 min in PBS and mounted on microscope slides with antifade mounting medium with DAPI (Vector Labs, H-1500-10). Fluorescent images were acquired using Zeiss Axiovert 200 inverted microscope with an Axiocam MRM camera.

### Quantification of phagocytosis using flow cytometry

BMDMs and BMDCs were plated overnight at 2 × 10^5^ cells per well of a 24-well plate. One hour before *D. hansenii* infection, medium was changed to 500 μL of complete RPMI with 1 μg/mL isotype control or neutralizing antibody at 37°C for 1 h. Neutralizing antibodies used in this study include anti-mDectin-1 (InvivoGen, mabg-mdect), anti-Mincle (InvivoGen, mabg-mmcl-2), anti-Dectin-2 (Bio-Rad, MCA2415GA), anti-CD11b (eBioscience, 14-0112-82), anti-CD18 (eBioscience, 14-0181-82), anti-CD11c (eBioscience, 14-0114-82), Rat IgG2a isotype control (InvivoGen, mabg2a-ctlrt-05), Armenian Hamster IgG Isotype (eBioscience, 16-4888-81), Rat IgG2b kappa isotype control (eBioscience, 16-4031-82), and Rat IgG2a kappa isotype control (eBioscience 16-4321-82). *D. hansenii*-yemCherry was cultured overnight at 30°C in YPD shaking at 240 rpm. Cells were pelleted, washed in sterile PBS, and counted using hemacytometer. Yeast cells (2 × 10^5^) were added per well for a multiplicity of infection of 1 yeast per macrophage. Plates were centrifuged at 300 × *g* for 5 min and incubated at 37°C.

One hour following infection with fluorescently labeled *D. hansenii*, plates were washed three times in PBS to remove extracellular yeast. Myeloid cells were scraped in MACS buffer (1% FBS, 2 mM EDTA, in PBS) using a miniature cell scraper (Thomas Scientific #1149M65). Cells were moved to a 96-well round bottom plate for staining. Myeloid cells were blocked with TruStain FcX (anti-mouse CD16/32, BioLegend) and stained with FITC-conjugated anti-CD45 (Clone 30-F11, BioLegend). Cells were resuspended in MACS buffer containing the viability stain Sytox Blue (ThermoFisher). Flow cytometry was conducted using an Attune NxT Flow Cytometer using a 96-well plate autosampler (ThermoFisher). Flow data were analyzed using FlowJo software (V10.8). In experiments using transgenic mCherry-expressing *D. hansenii*, the percentage of phagocytosis-positive cells was quantitated as percent of CD45+, Sytox Blue−, singlets containing mCherry compared to CD45+, Sytox Blue−, singlets total population.

### Generation of CRISPR mutants in RAW 264.7 cells

Lentiviral particles were generated using HEK293T cells transfected with second-generation lentiviral plasmids pMD2.G (Addgene #12259), psPAX2 (Addgene #12260), and pCW-Cas9 (Addgene #50661) (49). In brief, HEK293T cells at 80% confluence in a 10 cm dish were transfected using JetPrime (Polyplus 101000015) in 10 mL of complete DMEM. Media were harvested from the cells at 24 and 48 h post-transfection and pooled together. Lentiviral particles were concentrated using Peg-It solution (System Bioscience LV810A-1) according to manufacturer directions. RAW 264.7 cells were infected with the resulting pCW-Cas9 lentiviral particles for 2 days before selection using 3 μg/mL of puromycin (InvivoGen, ant-pr-1) in complete DMEM for 7 days, resulting in a doxycycline-inducible Cas9-expressing RAW 264.7 cell line (pCW-Cas9-RAW 264.7).

A scramble non-targeting control gRNA (GCTTAGTTACGCGTGGACGAAGG) was used as described elsewhere (50). Two gRNAs per gene were designed to target *Clec7A* (Dectin-1, gRNA1: GTACCGGAACTTACGAGTTGGGG, gRNA2: ATTCCTAAACCCACTGCAATGGG) and *Card9* (gRNA2: GCTCCTAGCGAACCGCGCCGAGG, gRNA3: GGGGATCGCGGGAATACGACAGG) using Chopchop (51). gRNAs were cloned into pLenti SpBsmBI sgRNA Hygro (Addgene #62205) as described (52). Lentiviral particles for each gRNA were generated as described above. pCW-Cas9-RAW 264.7 cells were transduced with scramble gRNA, both *Clec7A* gRNAs, or both *Card9* gRNAs. Two days following transduction, cells were selected using 200 μg/mL of hygromycin (InvivoGen, ant-hg-1) for 4 days. Cells were passaged and treated with 1 μM doxycycline to induce Cas9 expression and maintained in 50 μg/mL hygromycin for 1 week. Cells underwent single-cell cloning by plating limiting dilutions and allowing for expansion.

Cells were validated for protein knockout using immunoblot. Ten micrograms of protein lysate was submitted to SDS-PAGE, and membranes were blocked in BlockingOne buffer (Nacalai USA, 03953-66) prior to antibody incubation. Membranes were incubated with 1:1,000 dilution of rabbit anti-Dectin-1 antibody (CST, 23042S) or 1:1,000 rabbit anti-Card9 (CST, 12283) overnight at 4°C, washed, and incubated with 1:3,000 dilution of anti-rabbit HRP secondary antibody (CST, 7074) for 1 h at room temperature. β-actin was detected using 1:3,000 dilution of anti-β-actin HRP-conjugated primary antibody incubated for 1 h at room temperature (Proteintech, C825G26). Blots were incubated in chemiluminescent developing reagent (ThermoFisher #34580, BioRad #1705061), and signal was detected using a ChemiDoc (BioRad).

### Enzyme-linked immunosorbent assay

Four hours post-infection with *D. hansenii* as described above, supernatant was harvested and stored at −80°C until analysis. Mouse Tnf was detected per manufacturer’s directions (BioLegend #430901). TMB substrate (BioLegend 421101) was read on a BioTek Cytation5 plate reader measuring OD_450_ with OD_570_ background subtraction.

## Data Availability

Please direct requests for strains or materials used in this study to the corresponding authors, which will be made available through means of material transfer agreement. Data used to generate figures are deposited to Zenodo (53).

## SUPPLEMENTAL MATERIAL

Table S1 iai.00157-26-s0001.pdf Antibodies used in the cell surface receptor screen.

ASM does not own the copyrights to Supplemental Material that may be linked to, or accessed through, an article. The authors have granted ASM a non-exclusive, world-wide license to publish the Supplemental Material files. Please contact the corresponding author directly for reuse.
