# Supplementary material for: Innate immune recognition of Debaryomyces hansenii requires Dectin-1-Card9 signaling
Source: Infect Immun. Author manuscript; Available in PMC 2026 Sep 11. (PMC13552104; doi:10.1128/iai.00157-26)
Supplement: Supplementary file 1 [file NIHMS2208989-supplement-Supplementary_Material.pdf]

Table S1. Antibodies used in the cell surface receptor screen.

Antibody	Company	Catalog #
PE-anti mouse CD64(FcgRI)	Biolegend	161003
PE anti-mouse CD16	Biolegend	158003
PE anti-mouse CD16.2(FcgRIV)	Biolegend	149503
PE anti-mouse CD22 (FcgR2)	Biolegend	156403
PE anti-mouse CD21/CD35 (CR2/CR1)	Biolegend	123409
PE anti-mouse/human CD11b	Biolegend	101207
PE anti-mouse CD11c	Biolegend	117307
PE anti-mouse IFNAR-1	Biolegend	127311
PE anti-mouse IFN-γR β chain	Biolegend	113603
PE anti-mouse CD25	Biolegend	101903
PE anti-mouse CD124 (IL-4Rα)	Biolegend	144803
PE anti-mouse CD132 (common γ chain)	Biolegend	132305
PE anti-mouse CD126 (IL-6Rα chain)	Biolegend	115805
PE anti-mouse CD210 (IL-10 R)	Biolegend	112705
PE anti-mouse IL-33Rα (IL1RL1, ST2)	Biolegend	145303
PE anti-mouse CD120a (TNF R Type I/p55)	Biolegend	113003

PE anti-mouse Notch 1	Biolegend	130607
PE anti-mouse Notch 2	Biolegend	130707
PE anti-mouse Notch 3	Biolegend	130507
PE anti-mouse Notch 4	Biolegend	128407
PE anti-mouse CD282 (TLR2)	Biolegend	148603
PE anti-mouse CD284 (TLR4)	Biolegend	145403
PE anti-mouse CD285 (TLR5)	Biolegend	148107
PE anti-mouse CD40	Biolegend	157505
PE anti-mouse CD70	Biolegend	104605
PE anti-mouse CD274 (B7-H1, PD-L1)	Biolegend	124307
PE anti-mouse CD273 (B7-DC, PD-L2)	Biolegend	107206
PE anti-mouse GITR Ligand	Biolegend	120305
PE anti-mouse CD80	Biolegend	104707
PE anti-mouse CD86	Biolegend	159203
PE anti-mouse CD252 (OX40L)	Biolegend	108805
PE anti-mouse CD371 (CLEC12A)	Biolegend	143403
PE anti-mouse CD1d (CD1.1, Ly-38)	Biolegend	123509
PE anti-human/mouse/rat MR1	Biolegend	361105
PE anti-mouse CD206	Biolegend	141706
PE anti-mouse CD369 (Dectin-1)	Biolegend	144304
PE anti-mouse CD115 (CSF-1R)	Biolegend	135506

PE anti-mouse Tim-4	Biolegend	130005
PE anti-mouse CD270 (HVEM)	Biolegend	136303
PE anti-mouse CD153	Biolegend	106405
PE anti-mouse CD41	Biolegend	133906
PE anti-Mouse MHC Class II (IA/IE)	Tonbo	50-5321
PE anti-mouse CD31 (PECAM-1)	eBioscience	12-0311-82
PE anti-mouse SiglecF	BD	552126
PE anti-mouse/rat CD62P (P-Selectin)	Biolegend	148306
PE anti-mouse NK1.1	eBioscience	12-5941-81
PE Rat IgG2a, κ Isotype Ctrl	Biolegend	400507
PE Armenian Hamster IgG Isotype Ctrl	Biolegend	400908
PE Mouse IgG1, κ Isotype Ctrl	Biolegend	981804
PE Rat IgG2b, κ Isotype Ctrl	Biolegend	400608
PE Rat IgG1, κ Isotype Ctrl	Biolegend	400407
